# Recent advances in fabrication of dECM-based composite materials for skin tissue engineering

**DOI:** 10.3389/fbioe.2024.1348856

**Published:** 2024-01-23

**Authors:** Peiyao Xu, Jiutao Cao, Youyu Duan, Ranjith Kumar Kankala, Aizheng Chen

**Affiliations:** ^1^ Institute of Biomaterials and Tissue Engineering, Huaqiao University, Xiamen, Fujian, China; ^2^ Fujian Provincial Key Laboratory of Biochemical Technology (Huaqiao University), Xiamen, Fujian, China

**Keywords:** dECM, skin, wound healing, composite materials, tissue regeneration

## Abstract

Chronic wound management is an intractable medical and social problem, affecting the health of millions worldwide. Decellularized extracellular matrix (dECM)-based materials possess remarkable biological properties for tissue regeneration, which have been used as commercial products for skin regeneration in clinics. However, the complex external environment and the longer chronic wound-healing process hinder the application of pure dECM materials. dECM-based composite materials are constructed to promote the healing process of different wounds, showing noteworthy functions, such as anti-microbial activity and suitable degradability. Moreover, fabrication technologies for designing wound dressings with various forms have expanded the application of dECM-based composite materials. This review provides a summary of the recent fabrication technologies for building dECM-based composite materials, highlighting advances in dECM-based molded hydrogels, electrospun fibers, and bio-printed scaffolds in managing wounds. The associated challenges and prospects in the clinical application of dECM-based composite materials for wound healing are finally discussed.

## Introduction

Skin is the largest organ that acts as a protective barrier from the complex external environment (Li et al., 2022a). The health of the skin is often affected by various thermal, mechanical, and chemical hazards. Accordingly, wound management is a problem that humankind must face (He et al., 2023). Although the body possesses wound-healing ability by self-regulation with multiple cells, the wound-healing process is significantly affected by numerous factors (Cui et al., 2022). Bacterial infection, inflammation, and insufficient wound management may induce chronic wound infection, which frequently occurs in diabetes, cardiovascular disorders, and elderly patients (Las Heras et al., 2020; Xu et al., 2022b; Duan et al., 2023). Besides the loss of skin functionality in massive trauma, the scar formation also brought psychological and social challenges to patients (Fernandes et al., 2022).

Allogenic/xenogeneic tissue or organs have been used in clinical organ transplantation and skin tissue regeneration. However, the adverse immune rejection response induced by antigens of tissue or organs significantly increased the possibility of failure in organ transplantation (Wong et al., 2021). Fortunately, researchers have developed various decellularization methods to obtained decellularized extracellular matrix (dECM), including physical methods (freeze-thaw cycling and pressurization), chemical methods (bases and acids, detergents), and enzymes mediated biological methods (Golebiowska et al., 2024). During the decellularization process, the immunogenic cellular components of native tissue are removed, whereas the extracellular biomacromolecules and other tissue-specific bioactive molecules in extracellular matrix (ECM) are mostly preserved (Chen et al., 2023a; Xu et al., 2023). dECM could accelerate the wound healing process and enhance tissue repair by multiple cellular processes, including in motivating cell growth and proliferation, promoting re-epithelization and angiogenesis, and inhibiting inflammation (Wang et al., 2021). Decellularized extracellular matrix (dECM) based biomedical products (such as AlloDerm^®^ and Oasis^®^) have been used in the clinic as a promising substitute for regular wound dressings (Mostow et al., 2005; Callcut et al., 2006).

Although dECM tended to form hydrogels under 37°C to mimic tissue microenvironment, the unsuitable physicochemical properties and fast degradation rate of dECM still hinder the application (Chen et al., 2023b). The combination of natural and synthetic polymers with dECM could improve the mechanical properties, which has been used for promoting tissue regeneration (Zhang et al., 2022; Shen et al., 2023; Shi et al., 2023). Due to the risk of infections, developing antibacterial and effective dECM-based biomaterials for promoting wound healing is urgently needed. dECM hybrid scaffolds such as nanofiber films, sponges, hydrogels, and three-dimensional (3D) meshes with outstanding performance have been continuously constructed for tissue regeneration. The multiple approaches, including electrospinning, molding, and 3D printing, significantly expand the application of dECM-composite biomaterials.

**FIGURE 1:**
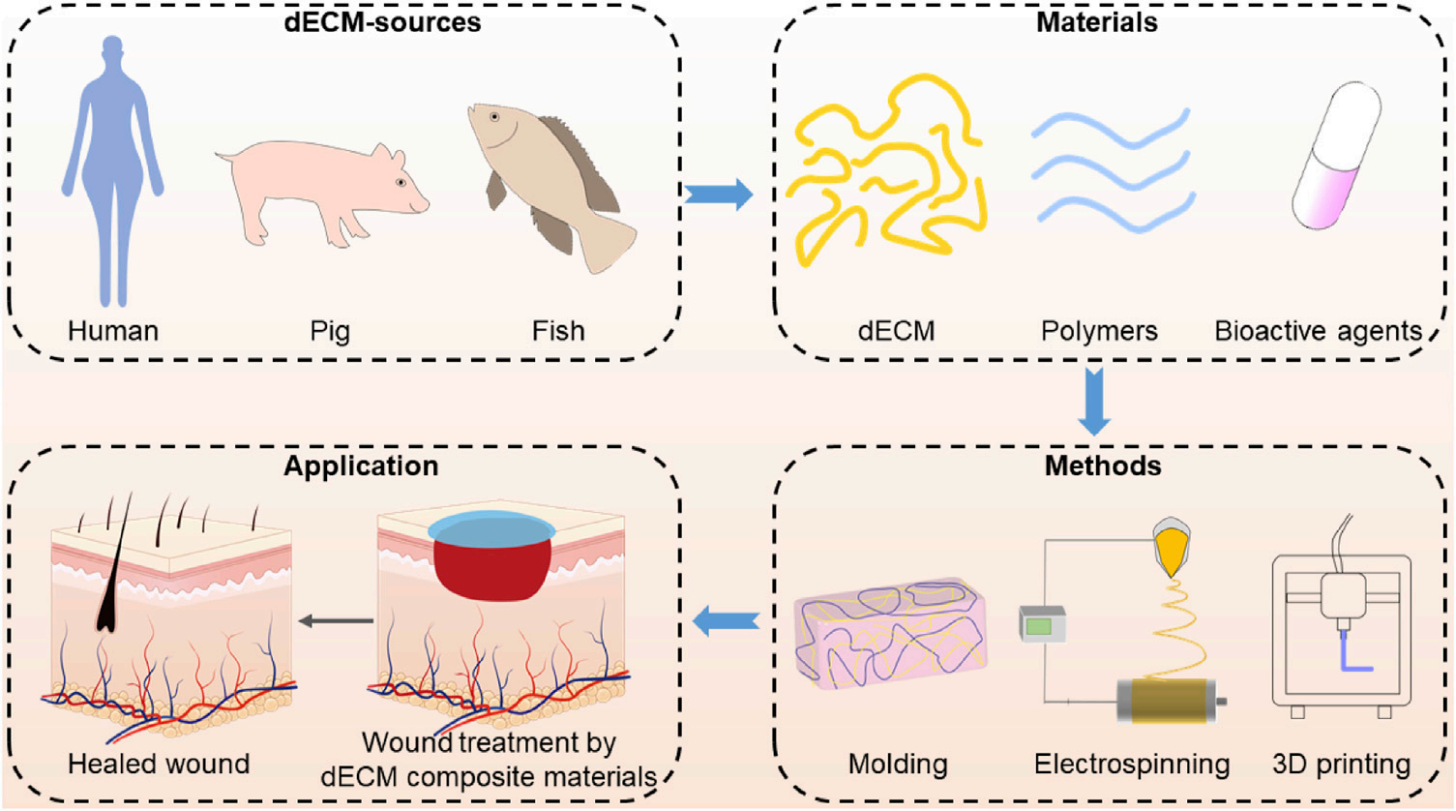
Fabrication of dECM composite and its use in wound healing application.

In this review, we focused on the combination of dECM-derived biomaterials with drugs or other biomaterials for skin tissue engineering. More specifically, we discussed the fabrication techniques for constructing dECM-composite scaffolds that are suitable for wound healing. Finally, we highlight the contemporary challenges and prospects of dECM-composite biomaterials in skin regeneration applications (Figure 1). This review aims to provide the comprehension of dECM-based composite biomaterials, guiding the researchers to choose suitable fabrication technologies and ultimately promoting the clinical translation of biomaterial in skin tissue engineering.

### Fabrication methods to build dECM-based composite materials

As mentioned, dECM-based composites have exhibited numerous outstanding outcomes in promoting skin regeneration. Therefore, it is crucial to build composite materials with appropriate morphologic forms, such as nanofiber, films, hydrogel, and 3D meshes, to manage different types of wounds. In this section, we have categorized recent literature based on fabrication technologies (including molding, electrospinning, and 3D printing) to build dECM-based composite wound dressing.

#### Molding

After decellularization, solubilized dECM powder could be manipulated to form a hydrogel using molding process after adjusting the suitable pH and physiological temperature, which may be based on the thermal cross-linking of collagen-induced self-assembly (Pati et al., 2014). Due to their excellent bioactivity, injectable feasibility, and thermal-sensitive properties, dECM-based hydrogels have shown attractive advantages in tissue regeneration (Zhang et al., 2021b). However, their rapid degradation and weak mechanical properties still pose a major challenge. Designing a dECM-based hydrogel system with suitable physical and chemical properties is required for expanding its application in the tissue regeneration field. For example, Xu et al. mixed gelatin, chitosan, and dECM to form composite hydrogel (dECM/Gel/CS), showing suitable compressive elastic modulus (≥482.17 kPa), excellent hydrophilicity, and appropriate degradability (Xu et al., 2021). The dECM/Gel/CS composite showed exceptional biocompatibility and stable antibacterial activity on *Escherichia coli* and *Staphylococcus aureus*. In another work, Zhang et al. successfully fabricated a photo-cross-linkable hydrogel based on dECM-methacrylate (dECM-MA) and gelatin methacryloyl (GelMA) as skin scaffolds (Zhang et al., 2021a). The mechanical strength and water absorption of dECM-MA/GelMA scaffolds were significantly improved compared to the GelMA and dECM groups. Moreover, immunohistochemical results of the wound area showed that the dECM-MA/GelMA group facilitated collagen deposition and promoted angiogenesis and re-epithelialization.

To avoid the secondary injuries of changing wound dressing, researchers have designed dECM-based hydrogel with appropriate adhesion for wound treatment. Wang et al. designed a composite hydrogel system that combines with o-nitrobenzene-modified gelatin-coated dECM to enhance the tissue adhesive property (Wang et al., 2023a). The composite scaffolds significantly accelerated the epidermal regeneration in the wound model by promoting angiogenesis and collagen fiber reformation. Likely, Bo et al. fabricated adipose-derived stem cells loaded hydrogel system made from a combination of dECM and o-nitrobenzene-modified hyaluronic acid (Bo et al., 2020). The photo-cross-linkable composite hydrogel exhibited good skin adhesive activity, which could apply to different kinds of skin defects with irregular forms. Moreover, the cells-loaded hybrid system significantly accelerated the wound closure rate, which was achieved by enhancing collagen deposition, promoting re-epithelialization, and promoting angiogenesis.

Besides improving the mechanical properties of dECM-based hydrogel wound dressing, numerous functional molecular or bioactive substances were combined with dECM to satisfy different wound microenvironments. For instance, Liu et al. developed a bioactive composite hydrogel by mixing dECM, asiaticoside-loaded polydopamine nanoparticles, and GelMA for wound healing (Liu et al., 2023). Compared with other groups, composite hydrogels exhibited higher regenerated hair follicle numbers, revealing the ability to promote scarless wound healing. Tang et al. combined dECM hydrogel with platelet-rich plasma derived from the human body to promote wound healing (Tang et al., 2022). The migration and tube formation assays on human umbilical vein endothelial cells (HUVECs) showed that the hydrogel could promote angiogenesis, and the immunofluorescence staining and polymerase chain reaction (PCR) analysis of RAW 264.7 cells indicated the capability of M2 macrophage polarization promotion. Moreover, the wound repair experiment of nude mice and porcine showed that hydrogel could significantly promote skin regeneration. Recently, Song et al. prepared adipose-derived mesenchymal stem cell-derived exosomes encapsulated dECM hydrogels (ECM@exo) for skin regeneration (Song et al., 2023). Culturing with human immortalized keratinocyte cells (HaCaT) and HUVECs, ECM@exo significantly enhanced cell proliferation, migration, and angiogenesis. *In vivo* evaluations investigated in normal and diabetic wound models showed that the ECM@exo could reduce the inflammatory factor (tumor necrosis factor-α (TNF-α) and interleukin-6 (IL-6)) expression. To controlling the releasement of bioactive factors, Xiao et al. proposed a dECM-MA hydrogel for the spatiotemporal delivery of macrophage-associated cytokine (Xiao et al., 2023). In detail, the dECM-MA hydrogel encapsulated pro-healing cytokines acted as the core, whereas the pro-inflammatory cytokines-loaded dECM-MA hydrogel was the sheath portion. Compared with single cytokines therapy, the spatiotemporal dual cytokines release hydrogel remarkably improved cell proliferation, increased the cell migratory rate, and induced higher collagen III/I expression in fibroblasts. Moreover, the hydrogel exhibited higher angiogenic effects *in vitro* and also accelerated skin reconstruction with high quality.

#### Electrospinning

Nanofibers or microfibers that are produced by electrospinning have emerged as potential biomedical materials for wound management (Memic et al., 2019). This electrospinning fibers-based wound dressing with large specific surfaces could fit different kinds of skin, providing a suitable environment for cell adhesion and growth (Wang et al., 2023b). The dECM-based electrospinning fibers have shown potential in skin repair due to the penetrating network structure that could mimic the ECM structure of natural skin. In contrast, dECM materials provide biochemical for tissue regeneration. By controlling the electrospun solution properties and process parameters, electrospun fibers combined with polymers and dECM have been utilized in tissue engineering applications (Krishtul et al., 2020; Li et al., 2022b).

Typically, dECM and polymers were dissolved in a suitable solution to fabricate composite electrospun fibers. For instance, Kim et al. prepared nanofibers of poly-D,L-lactide-co-glycolide (PLGA) and dECM with different concentrations (0.25%, 0.5% and 1%) (Kim et al., 2017). The increase in the dECM ratio significantly enhanced the mechanical strength of electrospun fibers, promoting the proliferation of rat granulation fibroblasts. Similarly, Kim et al. prepared nanofibrous electrospun hybrid scaffolds using heart dECM and poly (l-lactide-co-caprolactone) (PLCL) (Kim et al., 2018) as wound dressing materials. Compared with the gelatin-PLCL group, the prepared nanofibers effectively promoted angiogenesis and vessel maturation *in vitro,* accelerating wound healing by minimizing inflammation, promoting angiogenesis, and reducing scar *in vivo*. In addition, Gao et al. combined electrospinning with gas foaming techniques to fabricate 3D nanofiber scaffolds based on polycaprolactone (PCL) and dECM and loaded with *ε*-polylysine (Gao et al., 2023). The 3D nanofiber scaffolds with layer-like structures exhibited excellent thermal stability, hydrophilicity, and outstanding antibacterial activity. Moreover, *in vivo* results demonstrated that the nanofibers were suitable to accelerate wound healing by promoting CD31 expression and decreasing TNF-α expression.

Bioactive molecules, such as antibacterial drugs or particles, were also used in dECM-based nanofiber systems. For example, Chandika et al. fabricated usnic acid-enriched dECM-PCL nanofibrous scaffold for promoting wound healing (Chandika et al., 2022). Furthermore, the nanofiber scaffolds showed desirable anti-microbial properties and biofilm inhibition activities activity, indicating long-term usnic acid releasement for 14 days. In addition, Zhan et al. designed a dECM/PLCL multifunctional nanofibers platform loaded with copper and coated with chitosan (CTS@PLCL/DWJM@Cu) via a coaxial electrospinning approach (Zhan et al., 2022). The core-shell nanofiber exhibited initial releasement of chitosan for exerting early antibacterial activity, presenting sustained release copper to promote neovascularization. The core-shell nanofiber showed satisfactory mechanical properties, suitable hydrophilicity and protein absorption, and excellent inhibition efficacy of both *S. aureus* and *E. coli*. Overall, CTS@PLCL/DWJM@Cu significantly inhibited bacterial growth, promoted neovascularization, and created a microenvironment for collagen deposition, further promoting the wound healing of diabetic wounds.

Besides mixing dECM with polymer as an electrospinning solution, dECM composite materials with different electrospinning forms have been developed. For example, Tang et al. prepared PLGA nanofiber that cultured human adipose-derived stem cells (hADSCs) and then decellularized them to obtain dECM-PLGA meshes, promoting the wound healing process *in vitro* (Tang et al., 2019). In another investigation, Lee et al. developed human dermal fibroblasts loaded with electrospun PCL fibers to obtain a 3D multi-layered fibrous scaffold (Lee et al., 2016). In detail, the cell was cultured on PCL fiber for 4 days. After decellularization, the freeze-milling dECM-PCL powder was used to fabricate a fibrous scaffold by the electrohydrodynamic jet process. The SEM images showed that all scaffolds with micro-fibrous bundles dECM-PCL based scaffolds showed higher tensile modulus and faster water absorption rate than pure PCL-based scaffolds. Gholipourmalekabadi et al. fabricated bi-layer membranes by using human amniotic membrane dECM and silk fibroin nanofiber as 3D artificial skin (Gholipourmalekabadi et al., 2018). After being cultured with ADSCs for 7 days, the composite membranes significantly increased the expression of pro-angiogenesis factors compared with pure dECM.

#### 3D printing

3D printing has gained increasing attention in tissue regeneration due to the ability to prepare scaffolds with suitable 3D morphologies and structures by using computer-aided design (Wang et al., 2022b). 3D printing is a viable option to create suitable structures for matching the inhomogeneous wound site, which also mimics native skin tissue by combining biomaterials with cells (Tanfani et al., 2023). Due to the printability of dECM, researchers constructed different types of bioengineered 3D structures based on dECM, showing translational potential for promoting tissue repair (Hwang et al., 2022). However, the low viscosity and long gelation time may result in the poor shape fidelity of dECM hydrogel-based 3D structure (Yeleswarapu et al., 2023).

The addition of polymers in dECM-based bio-ink or mixing the dECM with other kinds of bio-ink has been employed to enhance the mechanical properties of the printed structure. Jorgensen et al. provided a hybrid bioink by combining fibrinogen and dECM for extrusion-based bioprinting (Jorgensen et al., 2020). Compared with the dECM hydrogel, the combination of fibrinogen demonstrated higher storage modulus and viscosity at low temperatures, which showed shear thinning properties and longer structural stability. Bashiri et al. prepared a bioink based on alginate/gelatin that loaded different human placental dECM concentrations (Bashiri et al., 2023). The addition of dECM could significantly improve the compressive strength and tensile strength of the 3D printing scaffold. Compared with the alginate/gelatin scaffold, the 3D printed scaffolds with 5% dECM significantly enhanced wound healing and pro-angiogenic gene expression. Fu et al. exploited a 3D-printed dECM-GelMA-HAMA scaffold that incorporated hADSCs as a skin substitute (Fu et al., 2023). The integrated 3D printed scaffold significantly promoted wound healing *in vivo*, relying on promoting angiogenesis, re-epithelialization, and collagen deposition, and inhibiting inflammatory response.

Additionally, there is an urgent need to design dECM-based biomaterial that inhibits bacterial infection for wound healing. Likewise, Xu et al. prepared a hybrid 3D printed tissue engineering scaffold composed of gelatin, quaternized chitosan, and dECM assembled with poly (ionic liquid)s (Xu et al., 2022a). By assembling poly (ionic liquid)s into the scaffold, the composite platform displayed a strong Gram-negative and Gram-positive bacteria inhibition rate, effectively preventing bacterial infection for a long time. Moreover, the composite scaffold demonstrated excellent hemostatic effect and hemocompatibility, thus showing an essential potential in skin engineering. Hu et al. reported a multifunctional dECM-MA-based 3D printing hydrogel scaffold embedded with copper epigallocatechin gallate nano-capsules for diabetic wound management (Hu et al., 2023). The cellular experiments indicated that the multifunctional 3D platform showed favorable anti-inflammatory capacity and good biocompatibility, which could accelerate vascularization and promote cell migration and invasion. *In vivo* experiments of the diabetic split-thickness skin graft model demonstrated that compared with commercial dECM groups, 3D-printed scaffolds significantly decreased the size of wound area with no obvious contracture. Moreover, 3D-printed scaffolds showed a similar ratio of collagen I/III in dermis and skin thickness to native tissue, indicating the potential tissue regeneration ability with non-scar in diabetic wound. In another work, Lin et al. prepared sodium alginate and dECM-MA as bio-ink to load with the curcumin and basic fibroblast growth factor (bFGF) for a 3D scaffold construction (Lin et al., 2023). The bFGF in scaffolds could significantly promote neovascularization, and the curcumin could inhibit bacterial growth, indicating a promising way to promote the bacterial infection wound for potential clinic applications. Nanoparticles with unique properties have been used in 3D structure construction, which may reinforce the mechanical and physicochemical properties of hydrogels or introduce the new biological functions of bio-ink (Chakraborty et al., 2021). Hu et al. fabricated 3D scaffolds by mixing dECM with mesoporous bioactive glass and exosomes as bioink to through extrusion-based printing technology (Hu et al., 2021). The composite hydrogel scaffolds showed a sustained release of exosomes for 14 days, and the addition of bioactive glass significantly promoted the cell proliferation, adhesion, angiogenesis. Moreover, the 3D wound dressing not only showed rapid hemostatic ability but also shortened wound closure rate and enhanced angiogenesis in diabetic wounds.

In nature, skin consists of epidermis, dermis, and hypodermis in order; relative cells were interspersed in ECM that supported the mechanical properties and resilience of skin (Daikuara et al., 2022). To simulate natural full-thickness skin with the epidermal and dermal compartment, Ahn et al. used a 3D bioprinting technology by sacrificial gelatin-assisted to build a full-thickness skin model, which was constructed by dECM-loaded human dermal fibroblast as dermis and sacrificial gelatin-loaded human epidermal keratinocyte as epidermal layer (Ahn et al., 2023). In another work, Jin et al. designed an artificial skin with a 3D structure, which was constructed by 20% GelMA as an epidermal layer, 1.5% porcine skin dECM as a dermis layer, and 10% GelMA as a vascular network (Jin et al., 2021). The *in vitro* results showed that the 3D-printing skin model provided a suitable microenvironment for cell proliferation and also supported epidermis reconstruction. In addition, the full-thickness wound test revealed that the 3D-printing skin substitute significantly enhanced the wound-healing process and promoted the synthesis of collagen III while inhibiting the excessive proliferation of collagen I.

## Conclusion and perspectives

Wound management remains a serious challenge burden on healthcare systems nowadays. Due to the limitations of dECM-based biomaterials alone in the wound healing process, the strategy of combining polymers or drug agents with dECM significantly enhanced the physicochemical property, showing great potential in skin regeneration. Moreover, the emergence of fabrication techniques and the breakthrough of apparatuses may provide a great way for dECM-based composite wound dressing in successful clinic translation. In this review, we have summarized the recent developments of dECM-based composite materials in wound healing, highlighting the significance of the fabrication method in the design and bio-function of those biomaterials.

As a promising field, some challenges remain in developing dECM-based composite wound dressing for clinic application. Various types of functional components with different mechanisms have been united with dECM-based biomaterials for promoting wound healing. However, due to the dynamic changes of the wound healing process, it is difficult to meet the requirements of the entire skin regeneration process. Moreover, the wound microenvironment is rather complicated and involves immune relation cells, pH, oxygen, enzymes, and microbiology, among others. Therefore, developing dECM-based wound dressing could respond to the wound microenvironment, and precise delivery of the bioactive agents on demand is a further direction.

Although dECM-based composite biomaterials with different forms are produced by various fabrication technologies as alternatives to skin grafts, skin is a complex organ that is composed of epidermal, dermal, and hypodermal, which contain different cell types and density, ECM composition, and microstructure. Fabrication of tissue-engineered skin substitutes with 3D micro-structure and the combination of relative cells are still challenging. Therefore, the integration of various fabrication technologies in building skin substitutes is promising. The cooperation of clinicians, scientists, and mechanical engineers may move the dECM-based biomaterial forward in the future.
